# Personal Experience with the Cholera Epidemic of 1852-53*Read before the Erie County, N. Y., Medical Society.

**Published:** 1884-11

**Authors:** E. T. Dorland


### Personal Experience with the Cholera Epidemic of 1852–53.*

By E. T. Dorland, M. D.

My only apology for presenting to this learned body a few thoughts on Asiatic cholera, is, that I was privileged with an opportunity of knowing something of the disease, from experience, where it prevailed in a very virulent form, viz., at the Erie County Almshouse during the serious epidemic of 1852–53. At that time I was resident physician of the institution and Dr. Pratt the attending physician. Professor Austin Flint and Dr. Winnie also visited the institution many times, in consultation, during the epidemic.

*Read before the Erie County, N. Y., Medical Society.

I assume that the profession are generally too well informed on the history of Asiatic cholera to make it proper for me to dilate upon it. We are all, I suppose, aware that the disease is endemic in certain parts of British India, where, from time to time, it assumes a virulent type, and is then apt to spread by favoring circumstances along the great lines of human intercourse, and so to extend over the world. The essential characteristics of all epidemics are the same, though differing from each other in the degree of virulence, necessitating, therefore, modifications of treatment according to the activity of the specific cause. Asiatic cholera was unknown in Europe prior to 1829 and 1830, although it had existed in India for many centuries.

The principal visitations of cholera in America were in the years 1829 and 1830, in 1848, in 1852, 1853 and 1854, and in 1873; this latter epidemic you were all, doubtless, familiar with, either by actual observation or by reading its history. Apropos of this last visitation I will state that a very full and careful history of facts concerning it may be found in the work of Dr. John M. Woodworth, Supervising Surgeon, United States Merchant Marine Hospital Service, entitled, "Cholera Epidemic of 1873 in the United States." Much valuable information is therein contained on the subject of sanitary science and disinfection. The symptoms of cholera you are all practically or theoretically acquainted with, but to those who have only a theoretical knowledge of the disease, I will say, that if you are ever called to treat a case of real Asiatic cholera you will never experience any difficulty thereafter in diagnosticating it. Such violent vomiting and purging, such enormous quantities of watery fluid ejected in so short a time, such severe and agonizing spasms, such rapid sinking, such lowering of the temperature, such early collapse and death, finds no parallel in any other disease ever yet known or recognized on the face of the earth. With no desire to magnify the importance of my observations, I thought, however, as many of the cases there were seen by Flint, Pratt and Winnie, and as several autopsies were made, and I, by request, made a record of them, a few points that impressed us as prominent and striking might be of some interest at this time. I find by the record then made, which I still have, that Professor Flint made two autopsies, and Dr. Winnie three, agreeing substantially in regard to the external and internal appearance of the bodies examined, so I will recite but two of them, one of Professor Flint's and one of Dr. Winnie's, and to avoid the possibility of errors in copying, will, with your permission, read from my old diary the original notes taken thirty-two years ago, just as they were written up at the time. The notes read as follows:

"June 5, 1852. Last night a young, robust German woman was taken with violent vomiting and purging; fluid ejected was of the rice water character; was pulseless; hands, feet and nose blue and shriveled; covered with cold sweat; intellect obtuse; great thirst; uneasiness at the epigastrium; respiration hurried; we applied mustard extensively, and made vigorous application of external heat by means of unslaked lime, etc., but she sank rapidly and died in about fourteen hours from the attack. This afternoon a young, healthy Irish woman was taken in the same manner, and in spite of prompt treatment by mustard and external heat, and the internal use of brandy, laudanum, camphor and capsicum, she is rapidly sinking.

"June 6, 1852. This morning, about 7 o'clock, the patient last referred to in my yesterday's diary died. Dr. Pride and myself thought both cases were real genuine Asiatic cholera, although no cases of the disease have yet been reported in this part of the country. We sent for Dr. Winnie to come and make a post mortem examination. He examined the body of the woman who died last, and found the following condition obtained: Capillaries of the stomach and intestines much congested, giving them a rosy tint; urinary bladder empty and gall bladder filled; but the most striking feature was the condition of the mucous membrane. This, of both stomach and bowels, was entirely decomposed and existed in a cream-like form, lining but not adhering to the muscular coat, and the bowels were filled with the same fluid evacuated before death. Dr. Winnie was of the opinion that the patient died of real Asiatic cholera."

In the same diary, under date of June 20, 1852, after describing the symptoms and treatment of several new cases of cholera, I find the following statement:

"John Devoy died about 3 o'clock this morning; sick seven hours; Dr. Flint was here about 10 o'clock and we made a post mortem on his body; the cadaver was wonderfully shriveled and skin mottled; his legs were almost black; rigor mortis had set in, and one leg was partially and very rigidly flexed. On opening the body the mucous membrane of the stomach and upper intestines were found greatly congested and swollen; many small and many large patches were seen in the stomach and intestines, owing, Dr. Flint says, to the epithelial coat having sloughed off at those points; the right side of the heart and the pulmonary artery were found full of very heavy, dark blood, but the left side contained but very little; the lungs appeared bloodless and collapsed. Dr. Flint said the cause of this was the blood being so thick, after losing nearly all its serum, it could no longer pass through the smaller pulmonary arteries; hence this great engorgement of the right side of the heart and pulmonary artery and the empty condition of the vessels of the lungs."

The cases referred to above were considered of special interest at the time as being the first cases of recognized cholera in Western New York in the epidemic of 1852, 1853 and 1854.

Pardon me now for inviting your attention to the cause of cholera. I have no expectation of enlightening you on the subject, but have some points to make in regard to it as related to the epidemic that came under my observation. As to the immediate cause and development of Asiatic cholera, I think the ground is fully covered by the six short propositions of Dr.

Woodworth in the work referred to. I will venture to quote them:

"1st. That Asiatic cholera is an infectious disease, resulting from an organic poison gaining entrance into the alimentary canal, acting primarily upon and destroying the intestinal epithelium.

"2d. That the active agents in the distribution of the cholera poison are the defecations of persons suffering from the disease in any of its stages. That in these defecations there exists an organic matter which is capable of reproducing the disease in the human organism to which it has gained access.

"3d. That cholera defecata coming in contact with, and drying upon any subject, such as articles of clothing, bedding and furniture, will retain, indefinitely, their power of infection. That in this manner a sure transmissibility of the cholera infection is effected, and that a distinct outbreak of the disease may occur by such means at great distances from the seat of the original infection.

"4th. That the specific poison, which produces the disease known as cholera, originates alone in India, and that, by virtue of its transmissibility through the persons of infected individuals or in the meshes of infected fabrics, the disease is carried to all quarters of the world.

"5th. That the respiratory and digestive organs are the avenues through which individual infection is accomplished. That through the atmosphere of infected localities cholera is frequently communicated to individuals. That water may become contaminated with the specific poison of cholera from the atmosphere, from surface washings, from neglected sewers, cesspools or privies, and that the use of water so infected will induce an outbreak of the disease; and,

"6th. That the virulence of a cholera demonstration, the contagion having been introduced into a community, is influenced by the hygienic condition of the population, and not by any geological formation upon which they may reside."

From what I have observed and read, I fully subscribe to the truthfulness of these several propositions. But as experience proves that the specific contagion may gain entrance to our land through our seaports in spite of the most vigilant attention to quarantine, it behooves us, I think, as physicians of an inland town, to devote our special attention to the predisposing or contributing causes, so that in case it should, unfortunately, break out in our midst, we may be found in the best possible condition to meet the much dreaded foe, and thus be enabled to greatly lessen the destruction it would otherwise accomplish. The two great and principal predisposing causes to cholera I believe to be filth and fear, and I hardly know which is the more potent. By filth I must be allowed to mean everything unwholesome, unclean, unhealthy and unsanitary. But as the question of preventive treatment, or the adoption of the most approved principles of sanitary science as safeguards against the introduction of this dread disease among us, will be the special subject, I suppose, of your deliberations this evening, it will not become me to enter largely into its discussion, but I desire simply to call your attention to a few facts as gathered from our observations in the epidemic referred to. We learned, by bitter experience, that cleanliness and disinfection meant something, and that to accomplish what it is capable of, it must, of necessity, be very thorough. The first year it was with us, viz., 1852, we thought we were doing all that was required in a sanitary way by having the floors of the sick wards washed often, by the free use of chloride of lime, and requiring the inmates to carry further away from the institution the slops, consisting of all the accumulated filth and garbage of the place, where it was dumped on the ground. That was about our sanitary status through the summer and autumn of the year 1852.

The only wonder is that in such a place of concentrated filth, such a pestilential breeding atmosphere, any of us survived to tell the tale. It was terribly fatal; nearly all attacked were swept away; but it must be borne in mind that the class of persons that fill our almshouses are usually physical wrecks, among which we should expect a far greater mortality. The following year, 1853, when it again broke out in Buffalo as well as at our institution, it found the almshouse and the city in about the same sanitary condition as in the preceding year. The people did not anticipate this recurrence, and had, therefore, done but little in a precautionary way. The disease at once assumed a far more virulent form than characterized it the year before. The people were thoroughly alarmed and clamored for protection. The medical profession went to work with a will and did what they could in the way of improving the general hygienic condition of the city. As a result of a medical conference, held at our institution, at which Prof. Flint was a conspicuous figure, very radical sanitary reforms were instituted. The necessity of general personal cleanliness was urged upon us, the importance of isolation of those affected, and the burning of all clothing soiled in the least with cholera defecata, and of all clothing worn by cholera patients. Debris of all kinds was ordered collected in heaps and destroyed by fire; all the outbuildings, including stables, chicken house, privies, etc., were ordered to be thoroughly cleansed and disinfected; all the cholera discharges were ordered carried a long way from the buildings and thrown into a deep trench, dug on purpose to receive them, and there mixed with a quantity of loose earth and chloride of lime; these and other precautionary rules were rigidly observed. The principal disinfectants used by us were chloride of lime, charcoal, fresh earth and carbolic acid. The effect of this radical sanitary revolution among us was surprising, and, surely, very gratifying. There was an immediate abatement of the malady, both in the numbers attacked and in the virulence of the disease. In fact, it soon became quite manageable. The mortality soon dropped from at least 75 per cent. down to about 25 per cent., all unquestionably due to greater cleanliness and disinfection, and the confidence and hope that it helped to inspire. I verily believe that what vaccination is to small-pox, hygienic regulations are to cholera, and that the people of our city may rest assured that a rigid observance of sanitary laws presents to this virulent disease a wall which is almost insurmountable.

If any further evidence were needed of the potency of filth as a predisposing cause of cholera, we have it in the history of the present epidemic as it has been raging recently in France. In towns where sanitary requirements are unknown or unheeded, and where the people are steeped in filth, cholera runs riot, while in a great and densely populated city like Paris, where intelligent hygienic regulations are enforced, the disease is comparatively unknown. You may all have noticed Harold Frederick's account, cabled to the *New York Times*, of his experience in the cholera infected towns of Marseilles, Toulon and Arles. A summary of his observations is given in a Buffalo daily, as follows: "From Frederick's dispatches we learn that the cholera is largely, if not wholly, due to the undisturbed presence and unnoticed accumulation of filth, the uncleanly habits of the people and their ignorance of sanitary laws. Panic has also played its part and swelled, very considerably, the mortality." As to the treatment of cholera, I will offer in this paper no suggestions, although we held very positive opinions on some points which we thought our experience justified, but the principles of which harmonize, I think, with the general principles of treatment laid down by most authors and teachers, so I will not trespass upon your time in presenting them.

A few observations now on the influence of fear as a predisposing cause of cholera, and I am done. I think I am safe in saying that there is no disease upon which fear exerts so potential an influence in the inducing of an attack as in that of cholera. Its effects upon the brain and nervous system somehow so lessen the vital force or resisting power that the system falls an easy prey to the operation of the specific cause if brought, by any means, in contact with it. We all believed that a large per cent. of the cases, in 1852, among the inmates of the almshouse, was directly due to the condition of fright and fear that prevailed among them, and we also believed that the feelings of hope and confidence inspired among them by our earnest and thorough sanitary regulations, in 1853, exerted a wonderful influence in checking the further ravages of the disease.

Several, through fright, ran away from the institution, and two of them, we knew, were taken with it before they got far away and died by the roadside. Our attending physicians were continually urging the officers of the institution not to think of danger, but to keep cheerful and courageous, and, with one exception, we maintained good cheer and hopefulness throughout, and with that one exception none of the officers or attendants suffered any serious attack of the disease. The exception mentioned was an assistant keeper; his case furnished very convincing evidence of the great power of fear in inducing an attack of the disease. He was brave and cheerful during the siege of 1852, and when it first broke out in 1853, he was nothing daunted. After about two weeks, however, it happened, one day that several were taken about simultaneously, and two died within a few hours. He then became greatly alarmed, and lost his courage, and gave way to a conviction that we should all die. He lost his appetite, could not sleep, became very despondent, and in four or five days was attacked very suddenly and very violently, and died within fourteen hours. I have no doubt that fear alone caused his attack, being in a place and in an atmosphere where the specific cause was at work. In conclusion, I will state that I regard the obligation of the medical profession to the people to consist of two things: First, recommending and insisting that the city be put in the best possible sanitary condition and kept so, as the only safeguard against its introduction amongst us, and, second, assuring the people that this matter properly attended to, there is, then, no occasion for fear or alarm; that if each individual will see to it that his own domicile is kept clean and wholesome, thus contributing his share to the desired sanitary condition of the city, realizing that a board ot health, unaided by individual effort, cannot accomplish everything, then may all rest in peace, with no anxieties in regard to any serious visitation of Asiatic cholera.

